# Sad Fetus Syndrome: Partial Molar Pregnancy with a Live Fetus

**DOI:** 10.7759/cureus.3175

**Published:** 2018-08-21

**Authors:** Syed Adeel Hassan, Ali Akhtar, Zia Ud Deen, Maham Khan, Somia Jamal, Sana Sohail, Abdur Rehman Azeem Dar, Muhammad Atif Masood Noori

**Affiliations:** 1 Internal Medicine, Dow University of Health Sciences, Karachi, PAK; 2 Internal Medicine, Combined Military Hospital, Islamabad, PAK; 3 Internal Medicine, Dow University of Health Sciences, New York, USA; 4 Radiology, Armed Forces Institute of Radiology and Imaging, Rawalpindi, PAK; 5 Internal Medicine, Abbasi shaheed hospital, Karachi, PAK; 6 Obstetrics and Gynecology, Khyber Teaching Hospital, Peshawar, PAK; 7 Internal Medicine, CMH, Lahore, PAK; 8 Internal Medicine, Dow Medical College, Karachi, PAK

**Keywords:** : partial mole, co-existing live fetus, sad fetus syndrome pakistan, maternal complications, gestational trophoblastic diseases

## Abstract

We report a case of partial mole and co-existing live fetus. This condition, uncommonly termed “sad fetus syndrome,” is a rare subclass of gestational trophoblastic disease. Our case involves a 25-year-old primigravid woman who presented to the outpatient department at 18 weeks of gestation with lower abdominal pain, vaginal spotting, and severe nausea. Ultrasound revealed a “grape bunch” appearance and a live, coexisting fetus. The patient underwent spontaneous abortion around the twentieth week of gestation. A postoperative ultrasound revealed an empty uterine cavity. She was discharged a few days afterward but was advised to follow up with serial repeat measurements of her beta-human chorionic gonadotropin levels.

## Introduction

Hydatidiform moles are classified into incomplete and complete moles. They are usually caused by abnormal conception and are characterized by edematous villi with a proliferation of trophoblasts. The initial clinical presentation common to all hydatidiform moles is elevated beta-human chorionic gonadotropin (β-hCG) levels with a rapidly enlarging uterus and the passage of grape-like masses from the vagina. Histological analysis of the grape-like mass reveals shedding of the hydropic villi. Hydatidiform mole subtypes are usually diagnosed using routine ultrasound assessments early in the first trimester [1]. A rare subclass of gestational trophoblastic disease is a partial mole with a co-existing live fetus—a condition sometimes called “sad fetus syndrome.” We report a case of a patient with sad fetus syndrome who presented to our tertiary care hospital.

## Case presentation

**Figure 1:**
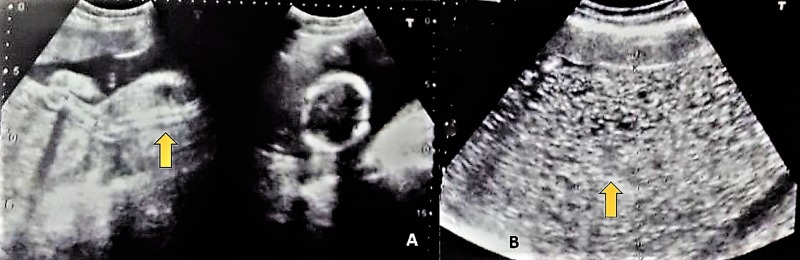
Ultrasound scans indicative of partial mole with a living fetus. A indicates enlarged placenta. B indicates partial mole with multiple cystic spaces in the placenta in a “snowstorm pattern.”

A 25-year-old woman reported to the outpatient department of our tertiary care hospital with lower abdominal pain, vaginal spotting, and severe nausea lasting 10 days. At the time of presentation, she was primigravid at 18 weeks and six days of gestation according to her last menstrual period. The pregnancy was a planned conception occurring within three months of marriage. The pregnancy was confirmed with a positive urine pregnancy test. Further questioning about menstrual history revealed cycles of four to five days every 28 to 30 days. Past medical, surgical or family history was unremarkable. On physical examination, she was alert and pale with a blood pressure of 150/90 mmHg and a pulse of 80 beats/minute. Her abdomen was soft, non-tender with a 32-week fundal height. A speculum examination revealed no active bleeding or discharge; the os was closed. Apart from the elicited anemic signs, the remainder of the physical examination findings were within normal limits. An ultrasound examination revealed a single live fetus of 18 weeks and two days in duration (Figures 1A-1B). We also noted a partial mole indicated by mass resembling a bunch of grapes and measuring 19 cm by 8 cm. The molar vascularity was insignificant on the Doppler evaluation. We ordered additional investigations through our hospital’s designated laboratory.

**Figure 2:**
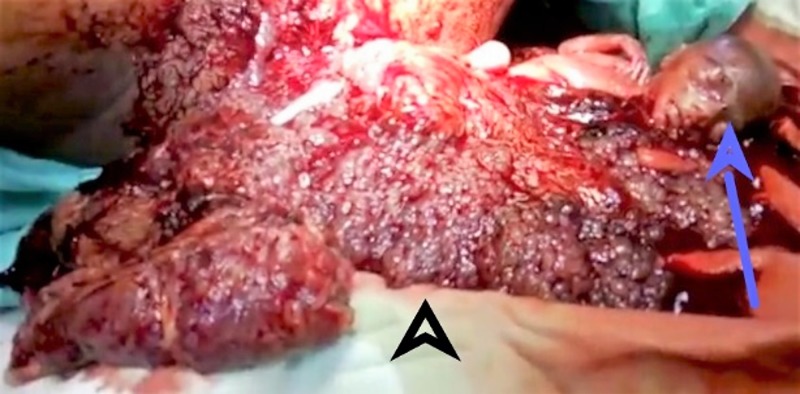
Photograph of the expelled contents. The male fetus is denoted by the blue arrow, and the partial molar tissue is denoted by the black arrowhead.

**Figure 3:**
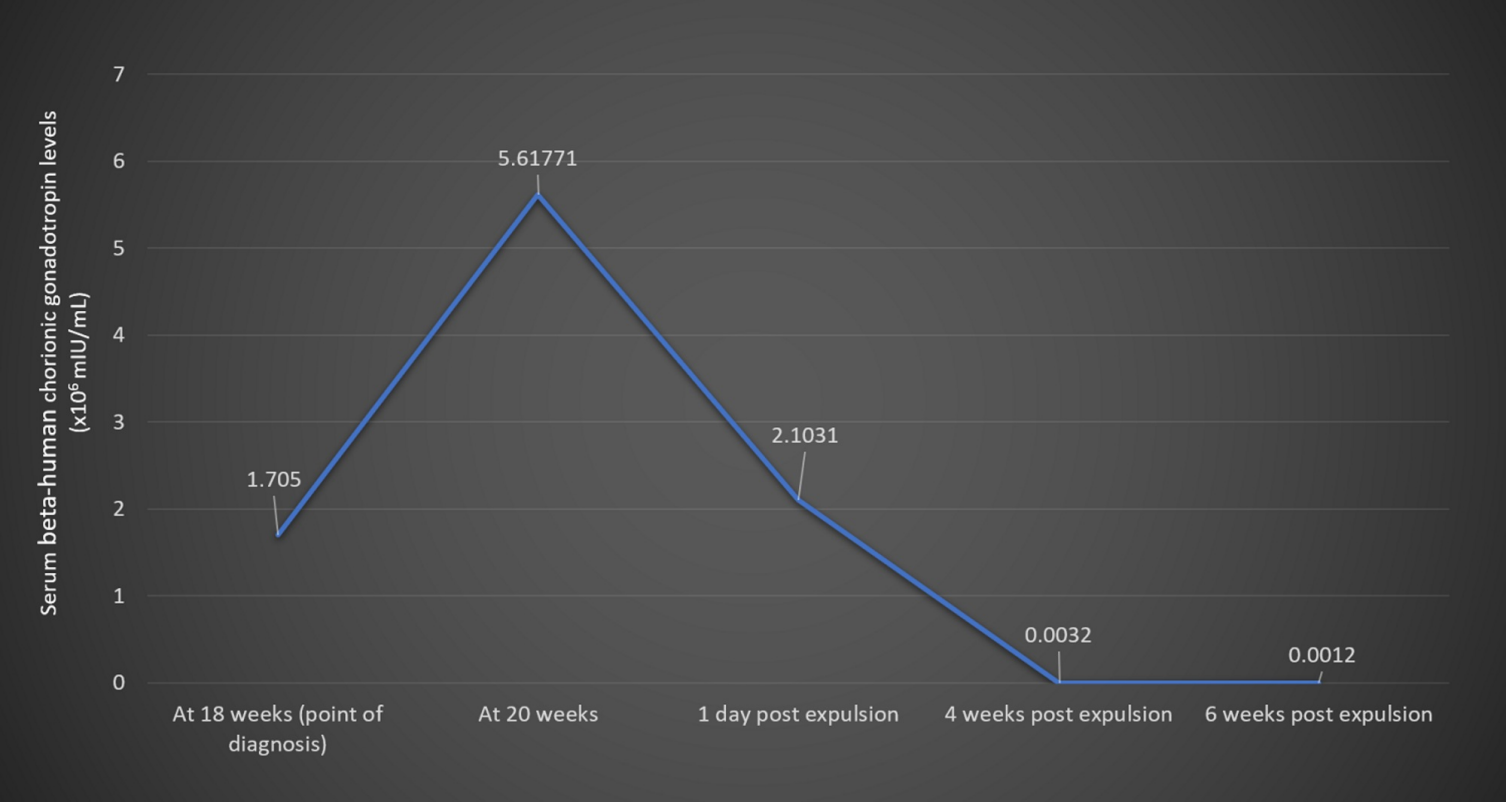
Clinical timeline with numerical progression of beta-human chorionic gonadotropin levels.

At 20 weeks of gestation, a repeat ultrasound revealed a single live fetus of 20 weeks duration with no gross fetal anomaly. A large mass with multiple short cysts was noted in the fundal region at the site of the placenta. We also noted evidence of a partial mole in the anterior fundal region of the uterus measuring 19.7 cm by 10.9 cm, bulging into the amniotic cavity and compressing the fetus. Repeat serial measurement of β-hCG at this point of pregnancy was 561,771 mIU/mL. At 20 weeks and two days, she reported concerns of a passage of grape-like vesicles, and spontaneous abortion followed. A live male fetus was expulsed with no grossly detectable anomalies, weighing 200 g, and placenta was expulsed weighing 100 g. After this, she expelled a large amount of molar tissue weighing approximately 500 g (Figure 2). The molar tissue was expelled intermittently via uterine contraction. After the expulsion of molar tissue, short-term general anesthesia was administered to the patient followed by suction curettage. An ultrasound after the procedure confirmed an empty uterine cavity. β-hCG levels were monitored post-termination. Approximately in one day the patient was discharged home, we noted a β-hCG level of 210,310 mIU/mL. Upon discharge, we instructed the patient to follow up with repeat serial measurements of her β-hCG levels. Four weeks following the abortion, her β-hCG levels were 320 mIU/mL, and six weeks following the abortion, β-hCG was undetectable. A plot of the change in the patient’s β-hCG levels over the course of her treatment is presented in Figure 3.

## Discussion

Sad fetus syndrome, also known as molar pregnancy with concomitant live fetus, is a rare phenomenon. Its incidence is reported at 0.005% to 0.01% of all pregnancies [1]. Molar pregnancies with a coexistent live fetus occur in three variations [2]. The most common variant includes a twin pregnancy with one fetus being healthy and the other being a complete mole. Less common is a twin pregnancy harboring a healthy fetus/placenta with a partial mole, and the rarest variant is a healthy single fetus with a partial molar placenta [2], as was the case with our patient. Our patient’s fundal height of 32 weeks at 18 weeks of gestation signified an exponential growth of the conceptus along with the substantial increase in the size of the partial mole (from 19 cm x 8 cm to 19.7 cm x 10.9 cm). Our patient’s β-hCG was initially measured at 170,500 mIU/mL, then increased to 561,771 mIU/mL—a staggering increase of 230% over a two-week period. Most cases result in termination and do not progress beyond 20 weeks of pregnancy [3]. Similarly, our patient underwent spontaneous abortion around the 20th week. In the event of a single normal fetus with partial mole, fetal survival depends upon the normal karyotype of the fetus, smaller molar placenta, rate of molar degeneration, the absence of anemia, and coexisting maternal complications such as pre-eclampsia, thyrotoxicosis, and vaginal bleeding [1]. The larger size of the molar placenta may have been an unfavorable prognostic factor for our patient. Maternal age is an important factor among many others. A study conducted by Al-Mulhim reports that hydatidiform cases occurred more prominently in mothers aged 21 to 35 years and was lowest in patients younger than 20 years [4]. Common presentations include bleeding per vaginam (90%), anemia (51.1%), hyperemesis gravidarum (28.9%), pregnancy-associated hypertension (12.2%), and thyrotoxicosis (3%) [4]. Our case was consistent with the literature, given she experienced bleeding per vaginam, anemia, and emesis. Such pregnancies are usually complicated by hemorrhage, sepsis, thyrotoxicosis, invasive mole, and choriocarcinoma. Parveen et al. reported a case of partial mole with a live fetus with term gestation that resulted in a live, healthy fetus [5]. The management of a partial mole with coexistent fetus remains a challenge due to the wide array of complications associated with the condition. Amniocentesis remains the diagnostic test of choice, and termination of the pregnancy may be required in due course.

## Conclusions

Molar pregnancy with a coexistent live fetus is a rare and challenging condition. Common clinical presentations that should alert the clinician to this rare condition include bleeding per vaginam, anemia, hyperemesis gravidarum, hypertension, thyrotoxicosis, and uterine size disproportionate to uterine age. The management of a partial mole pregnancy with a coexistent fetus is especially challenging due to the wide array of complications, the most common of which are severe hemorrhage, sepsis, thyrotoxicosis, and coexistent mole/choriocarcinoma. Ultrasound examination and β-hCG level measurements have proved to be efficient in tracking the clinical progression of the condition. Amniocentesis is the diagnostic test of choice, and terminating the pregnancy may be required.
